# Lamivudine plus adefovir is a good option for chronic hepatitis B patients with viral relapse after cessation of lamivudine treatment

**DOI:** 10.1186/1743-422X-8-388

**Published:** 2011-08-04

**Authors:** Zhao Wang, Xiao-Ling Wu, Wei-Zheng Zeng, Hui Xu, Yong Zhang, Jian-Ping Qin, Ming-De Jiang

**Affiliations:** 1 Department of Digestion, Chengdu Military General Hospital, Sichuan, People's Republic of China

**Keywords:** ADV plus LAM, LAM alone, retreatment, chronic hepatitis B

## Abstract

**Aim:**

Currently, there is no consensus on the retreatment recommendation of chronic hepatitis B (CHB) patients with viral rebound after cessation of treatment. In the search of reasonable treatment, we compared the efficacy and safety of adefovir (ADV) plus lamivudine (LAM) and LAM alone for the retreatment of patients with viral relapse but without genotypic resistance after cessation of LAM.

**Methods:**

This is a prospective controlled study, and a total of 53 hepatitis B e antigen (HBeAg)-positive patients with viral rebound but without resistance were received either LAM plus ADV or LAM alone treatment.

**Results:**

After 1-year treatment, more patients who received LAM plus ADV than those who received LAM alone had ALT normalization (84% versus 53.6%, P = 0.018) or HBV DNA levels below 1000 copies/mL (80% versus 42.9%, P < 0.006). Seven patients receiving LAM plus ADV had HBeAg seroconversion, as compared with 0 in patients receiving ALM alone (28% versus 0%, P = 0.003). During 1-year retreatment, five patients receiving LAM alone had virological breakthrough and all of them had LAM resistance strains (rtM204V/I), while no LAM- or ADV- associated resistance strains were detected in patients receiving LAM plus ADV. All patients receiving LAM plus ADV were well tolerated, and no serious side effects were noted.

**Conclusions:**

Patients treated with LAM plus ADV exhibited significantly greater virological, biochemical and serological responses compared with LAM alone. These data suggested that combination of LAM plus ADV would be a good option for the retreatment of CHB patients with viral relapse after cessation of LAM.

## Background

Estimated 350~400 million individuals worldwide are chronically infected with hepatitis B virus (HBV)[1], and chronic hepatitis B(CHB) can progress to cirrhosis, hepatocellular carcinoma (HCC), and death[2]. Antiviral therapy is used in CHB to minimize the liver damage and progression of disease[3], but total eradication of the HBV is seldom achieved by current treatment, especially for patients who infected HBV in early childhood.

Nucleos(t)ide analogs approved for treatment of CHB include lamivudine (LAM), adefovir (ADV), entecavir, telbivudine and tenofovir, and they have been considered to have a good effect in suppressing virus replication with few side effects[4]. In general, HBeAg seroconversion is an important end point for HBeAg-positive patients, which is associated with sustained remission[5,6]; and international guidelines suggested that early withdrawal of antiviral therapy, without reaching sustainable serological response, would lead to a very high risk of relapse[4]. In fact, a wealth of clinical experience also suggested the problem of viral relapse after drug withdrawal could not be ignored [7-9]. Some investigators had reported that viral load rebound is common in CHB patients receiving nucleos(t)ide analogs and the relapse rates would be high to 70%[8,10,11]. However, nearly 40% of the relapses or virological breakthroughs were not related to antiviral drug resistance. For example, due to the poverty, many CHB patients in Asia cannot afford their long-term medication, and they always terminated their treatment just when reaching virological response irrespective of the occurrence of HBeAg seroconversion. How to therapy those patients with viral relapse has become an urgent problem that we have to face[12]. Unfortunately, there is no consensus on the retreatment of viral relapse with or without resistance after cessation of nucleos(t)ide analogs treatment. And it is still unknown whether there is significant difference between original drug monotherapy or combined with other drugs without cross-resistance.

Current study was designed to compare the efficacy and safety of LAM plus ADV with that of LAM alone again in CHB patients with viral relapse after cessation of previous LAM treatment.

## Results

### Characteristics of the study patients

**Figure 1:**
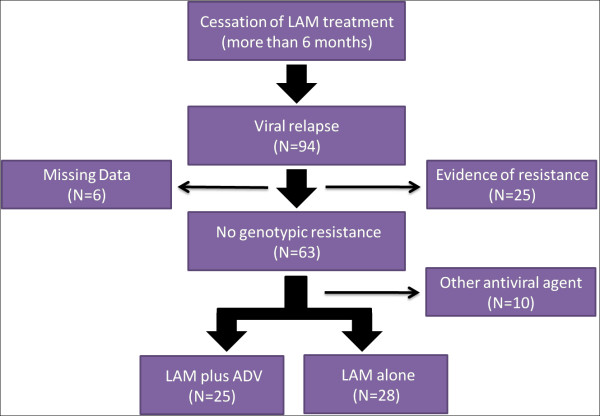
**Flow chart of patients selected in this study**.

A total of 53 HBeAg-positive CHB patients were analyzed in the study, which comprised of 25 patients in LAM plus ADV and 28 patients in LAM alone retreatment (Figure 1). The demographic and disease parameters were well matched at baseline of retreatment between two groups, which were described in detail in table 1.

**Table 1:** Baseline Characteristic of the study population

Characteristic	LAM plus ADV (N = 25)	LAM (N = 28)	P value
**Male sex -no. (%)**	20(80%)	21(75%)	0.664
Age - yr*	35.48	36.81	0.176
ALT - IU/L*	149.52	136.25	0.247
HBV DNA - log10 copies/ml*	5.89	5.96	0.812
Previous LAM therapy- yr*	3.60	3.88	0.115

*Expressed as mean

### Biochemical response

**Figure 2:**
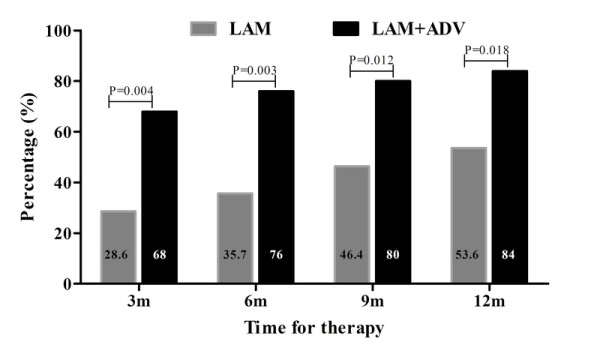
**Biochemical response rates of patients receiving ADV+LAM or LAM alone during the first year of retreatment**.

Serum ALT levels declined in both treatment groups, but ALT level was normalized in a higher proportion of LAM plus ADV than LAM alone retreatment in total. At 6 and 12 months after retreatment, normal ALT was achieved in 76% (19/25) and 84% (21/25) of patients receiving LAM plus ADV, and in 35.7%(10/28) and 53.6% (15/28) of patients receiving LAM alone, respectively (Figure 2). The difference in ALT normalization between two groups was significant at year 1(84% vs 53.6%, p = 0.018).

### Virological response

**Figure 3:**
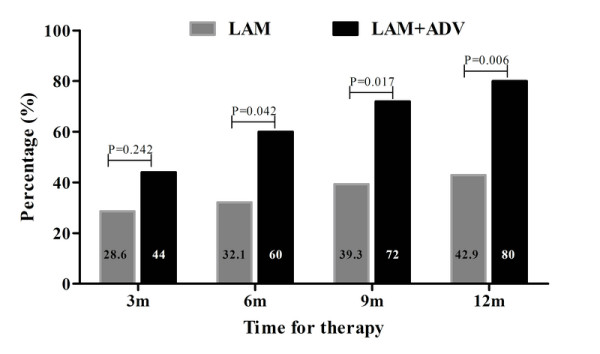
**Virological response rates of patients receiving ADV+LAM or LAM alone during the first year of retreatment**.

The reduction in serum HBV DNA levels from baseline of retreatment during the first year of observation period was greater in LAM plus ADV than in LAM alone retreatment (Figure 3). At 6 months, the proportion of patients with virological response (HBV DNA < 3 log copies/ml) was achieved in 60% (15/25) of patients receiving LAM plus ADV retreatment as compared to 32.1% (9/28) of patients receiving LAM alone. At 12 months, the proportion of patients with virological response was achieved in 80% (20/25) of patients receiving LAM plus ADV as compared to 42.9% (12/28) of patients receiving LAM alone. The difference in virological response between two groups was statistic significant at 6 and12 months, respectively (P = 0.042 for month 6 and P = 0.006 for month 12).

### HBeAg response

**Figure 4:**
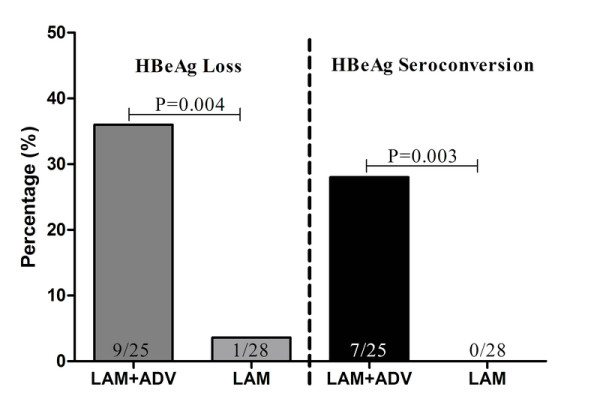
**HBeAg loss and seroconversion rates of patients receiving ADV+LAM or LAM alone during the first year of retreatment**.

More patients receiving LAM plus ADV retreatment had HBeAg seroconversion as compared to patients receiving LAM alone retreatment at month 12 (Figure 4), and the difference in HBeAg seroconversion between two groups was significant (7/25 versus 0/28, P = 0.003). Additionally, significant more patients receiving LAM plus ADV obtained HBeAg loss as compared to patients receiving LAM alone at month 12(9/25 versus 1/28, P = 0.004)(Figure 4).

### Breakthrough and resistance

Viral breakthrough was found in 5 patients receiving LAM alone retreatment during the 1-year observation period, and the mutations of rtM204V were found in 3 patients and rtM204I were found in 2 patients at year 1 of retreatment. In LAM plus ADV retreatment patients, no viral breakthrough were found and no LAM or ADV associated mutations were detected.

### Safety

Majority of patients were well tolerated in two retreatment groups. Among patients receiving LAM plus ADV, only1 patient had serum creatinine slightly increasing but there was no discontinuation due to this adverse event. Elevations in ALT were observed less frequently in LAM plus ADV group than LAM alone in group. ALT flares during the first year of treatment were in 5 patients with LAM alone and 1 patient with LAM plus ADV. In LAM plus ADV group, all the ALT flares were associated with toil; while in LAM alone retreatment group, all the ALT flares were associated with HBV DNA flares. Between two groups, none hepatocellular carcinoma was reported.

## Discussion

Although LAM is not recommended as first line therapy for CHB in the American Association for Study of Liver Disease (AASLD) CHB guidelines[4], and the European Association for Study of Liver (EASL) CHB guidelines[13], which recommend oral nucleosides or nucleotides with high genetic barrier (entecavir and tenofovir) as first line monotherapy, LAM is still widely used in Asia due to cost constraints[14]. Thus, it is necessary for us to concern about the re-treatment issues of patients with viral relapse after discontinuation of LAM monotherapy. Unfortunately, the retreatment options for viral relapse are still in doubt. In clinical practice, a variety of retreatment strategies have been applied, including back to their original drug, switch to another drugs, or two or more drugs in combination.

Theoretically, if there were no evidence of antiviral resistance of original agent, it seemed reasonable to back to their original drug for retreatment. However, this hypothesis was recently questioned by clinicians. Evidence already had showed that HBV exists in form of quasi-species in patients with chronic hepatitis B[15], and because of the sensitivity and specificity limitation of detection, drug-resistance strains sometimes could not be detected in time, especially when its proportion was less than 20% in the pool of viral quasi-species[16,17]. Thus, it is not difficult to conclude that before the drug-resistance strains reach a high number, they may not be detected in patients with viral relapse after cessation of antiviral treatment[16]. But it is worth mentioning that those inferior strains would be easily developed to predominant strains with antiviral resistance by positive selection of antiviral agents [18,19]. Taking into account of the above reasons, when viral relapse occurred after cessation of treatment, switch to agents with high genetic barrier or combination of two or more agents without cross-resistance may be good options for preventing the occurrence of drug resistance[9,20].

In this study, we explored and compared retreatment options for chronic hepatitis B patients with viral relapse after cessation of LAM. After 1 year retreatment, the proportion of undetectable HBV DNA was achieved in 80% of patients receiving LAM plus ADV as compared to 42.9% of patients receiving LAM alone. Moreover, higher proportion of ALT normalization and HBeAg seroconversion were also reached in LAM plus ADV retreatment group. Several other studies also had reported that the combination of LAM and ADV could lead to effective viral suppression in most cases after development of viral breakthrough due to LAM monotherapy[21,22]; and patients receiving LAM plus ADV combination therapy have a lower risk of developing genotypic resistance to ADV[22]. In our study, patients receiving combination therapy of LAM and ADV were well tolerated, and no viral breakthrough was reported and no LAM- or ADV-associated resistant strains were detected. In contrast, among 28 patients receiving LAM alone, five patients experienced a viral breakthrough and LAM-associated resistant strains were detected in all of them. Those findings gave us another hint that the combination of LAM plus ADV was associated with lower antiviral resistance compared with LAM alone. In fact, for some special populations with chronic hepatitis B, the combination of agents without cross-resistance had been clearly put forward by authoritative guidelines[4,13], and data from many clinical observational trials also suggested combination therapy would bring more benefits to nucleosides or nucleotides-refractory CHB patients.

At present, majority of clinical studies on LAM plus ADV combination therapy were for HBeAg-negative patients, and few studies on HBeAg-positive patients reported the combination of these agents had no synergistic on HBeAg seroconversion. But in our study, among 25 patients receiving combination therapy of LAM plus ADV, we delighted to found that 9 patients obtained HBeAg loss and 7 patients obtained HBeAg seroconversion, which were significant higher to that of patients receiving LAM alone. However, because of the limited sample size and short observation time, we currently cannot conclude that the combination of ADV plus LAM could increase the rate of HBeAg seroconversion as compared to single nucleoside or nucleotide analogue.

In conclusion, the current study showed that CHB patients with viral relapse after cessation of LAM retreated by LAM plus ADV exhibited significantly greater virological, biochemical and serological responses compared with LAM alone. These findings indicated that combination of LAM plus ADV would be a good option for the retreatment of CHB patients with viral relapse after cessation of LAM.

## Methods

### Patients

Outpatients from Chengdu Military General Hospital, with viral relapse after cessation of LAM treatment were screened, of who included in this study should also meet the following criteria: serum hepatitis B e antigen (HBeAg) positive; serum HBV DNA load above 1000 copies/ml; alanine aminotransferase (ALT) levels between 2 and 10 times the upper normal level. And patients who fulfilled one of the following criteria should be excluded: evidence of LAM associated resistance (rtM204V or rtM204I); presence of serum antibodies against hepatitis C virus (HCV), or human immunodeficiency virus (HIV); breast-feeding, pregnancy or inadequate contraceptive measures; other acquired or inherited causes of liver disease; coexisting serious medical disease; advanced liver disease(including decompensated cirrhosis with ascites, severe hepatitis, and hepatic carcinoma).

### Study design

This is a prospective controlled study, aimed to evaluate and compare the efficacy of LAM plus ADV with that of LAM alone retreatment in patients with viral relapse after cessation of LAM antiviral therapy. All included patients were administered 100 mg of LAM plus 10 mg of ADV (GlaxoSmithKline) or 100 mg of LAM alone daily according to patients' choice, and they were followed up in outpatient clinic of hospital. Clinical data were collected at baseline, and every 3 months after retreatment. The primary efficacy outcomes were ALT normalization, reduction in HBV DNA, and seroconversion of HBeAg. Second efficacy outcome was antiviral resistance.

This study was approved by Chengdu Military General Hospital's institutional review board and was conducted in accordance with the 1975 Declaration of Helsinki.

### Serum assay methodology

HBeAg and serum HBV DNA were measured with the use of Enzyme-Linked Immunosorbent Assay (InTec Technology Co., Ltd., Xiamen, China) and real-time PCR-based quantification assays(DA AN gene Co., Ltd., Guangzhou, China) according to the manufacturer's instructions, respectively. Serum ALT and Creatinine(Cr) were measured using automatic biochemistry analyzer according to standard laboratory procedures. Antiviral resistance was analyzed using PCR sequencing assay if HBV DNA no less than 1000 copies/mL after 1-year antiviral treatment.

### Definition

Biochemical response was defined as a decrease in serum ALT to within the normal range. Virological response was defined as a decrease in serum HBV DNA to undetectable levels by PCR assays (<1000 copies/mL). HBeAg response was defined as a loss or seroconversion of HBeAg in patients who were initially HBeAg positive. Virological breakthrough was defined as an increase in serum HBV DNA by 1 log10 (10-fold) above nadir, or to detectable level (≥ 1000 copies/mL) after achieving virological response during retreatment. Viral relapse was defined as an increase in serum HBV DNA to detectable level after achieving virological response.

### Statistical analysis

Quantitative variables were expressed as mean, and categorical variables were presented as counts and percentages, and HBV DNA levels were presented as log transformation. Comparisons between groups of quantitative and qualitative variables were performed using the student t test and Chi-square test (or Fisher's exact test), respectively. A P-value of less than 0.05 (two-tailed) was considered to indicate a significant difference. All statistical analyses were performed using the SPSS software package version 13.0 (SPSS Inc., Chicago, IL).

## Competing interests

The authors declare that they have no competing interests.

## Authors' contributions

MDJ conceived the study and revised the manuscript critically for important intellectual content. ZW, XLW and WZZ made substantial contributions to its design, acquisition, analysis and interpretation of data. XH, YZ, JPQ participated in the design, analysis and interpretation of data. All authors read and approved the final manuscript.
